# Complete rectal prolapse presenting with colorectal cancer

**DOI:** 10.1515/iss-2023-0014

**Published:** 2023-10-27

**Authors:** Aristoteles Perrakis, Frank Meyer, Hubert Scheidbach

**Affiliations:** Department of General, Abdominal, Vascular and Transplant Surgery, University Hospital, Magdeburg, Germany; Department of General and Abdominal Surgery, St. Theresien Krankenhaus, Nürnberg, Germany

**Keywords:** rectal prolapse, colorectal cancer, coincidence, case management

## Abstract

**Objectives:**

Rectal prolapse is defined as prolapse of all layers of rectal wallout of the anal sphincter. The aim was to (i) describe the extremely rare combination of a rectal prolapse with colon cancer in an older female patient, (ii) comment on management-specific aspects and (iii) derive some generalizing recommendations by means of a scientific case report and based on the case-specific experiences related to the clinical management and current references from the medical literature.

**Case presentation:**

A 69-year old female patient with cancer of the sigmoid colon at a manifest rectal prolapse was diagnosed. Literature search (using the data bank of “PubMed”) resulted in only six patients (the majority of them were females) with the coincidence of rectal prolapse and rectal or colon cancer have been reported so far.

**Conclusions:**

A patient with a manifest rectal prolapse needs always to undergo colonoscopy and – in case of an ulcer – histological investigation of representative biopsies.

## Introduction

Rectal prolapse is defined as prolapse of all layers of rectal wallout of the anal sphincter; it needs to be strictly distinguished from anal prolapse. Rectal prolapse occurs preferentially in female patients older than 50 years of age. The incidence amounts 2.5 cases per 100,000 inhabitants and is – therefore – relatively low [1]. Based on this background, combination of rectal prolapse with colorectal cancer is extremely rare, which has been only seldomly described in the world-wide scientific literature.

The aim of the manuscript was to describe the extraordinarily rare combination of a rectal prolapse with colon cancer at the externalized segment of the sigmoid colon in an older female patient by means of a scientific “Case Report” based on selected references from the literature and own clinical experiences obtained in the diagnostic and therapeutic management of the case including the attempt of a few generalizing comments and recommendations on the subject.

## Case report

**Medical history:** A 69-year old female patient was transferred by her family practitioner to the Department of Surgery after the colleague had repositioned the first manifestation of a rectal prolapse two days ago. At this appointment, prolapse could not be provoked despite several attempts. Therefore, the patient was recommended to rearrange immediately an appointment in case of a recurrent rectal prolapse.

**Figure 1:**
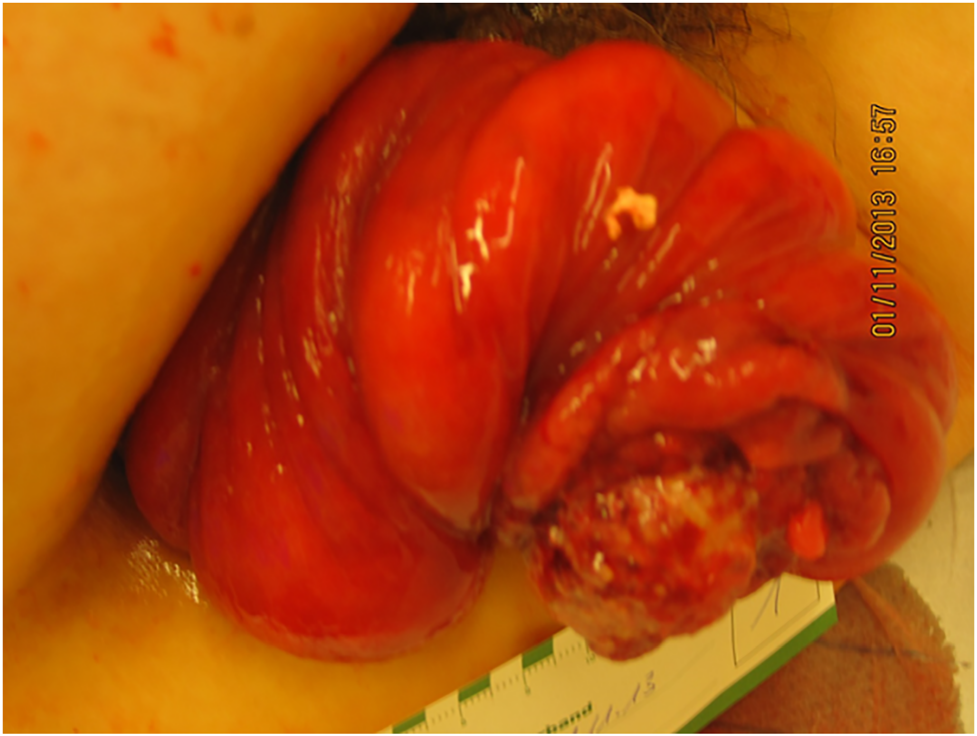
Clinical manifestation of rectal prolapse with exulcerated rectal adenocarcinoma at the externalized rectal mucosa.

**Pretherapeutic course:** Three weeks later, patient presented with typical rectal prolapse combined with a circular ulcer at the externalized segment of the rectum with 3 cm of size (Figure 1).

Further four days later, patient came again to the outpatient clinic with a manifest re-prolapse of the rectum after several episodes of rectal relapses on the former days.

**Decision(-making):** Therefore, the patient wished to be admitted to the hospital.

**Diagnostics:** Since the finding of the histological investigation of ulcer biopsy taken the days before described adenocarcinoma, diagnostic profile was completed; abdominal CT scan revealed rectal cancer with hepatic metastases while colonoscopy demonstrated cancer of the sigmoid colon at 35 cm above anocutaneous line.

**Diagnosis:** Rare combination of a rectal prolapse with colon cancer at the externalized segment of the sigmoid colon.

**Therapeutic approach:** The patient was prepared for surgical intervention. As part of the laparoscopic anterior resection of the sigmoid colon and upper rectum, diffuse metastases of the liver were punctured and diagnosed by means of fresh frozen section. Histopathological investigation confirmed hepatic metastases of colon cancer.

**Clinical course:** Postoperative course was uneventful.

**Further measures:** Prior to discharge of the patient, she underwent implantation of a port-a-cath for planned chemotherapy with palliative intention.

## Discussion

Rectal prolapse associated with colorectal cancer is considered a rare coincidence as revealed in the international scientific literature. In particular, only six patients – five of them were women – have been described so far, each of them presented as medical case report with the finding of a colorectal cancer at the mucosa of the externalized segment of the colon (Table 1) [2], [3], [4], [5], [6], [7].

**Table 1:** List of published cases with the combination of rectal prolapse and colorectal cancer (chronological order).

Author	Year	Sex	Age, years	Tumor site
Cougard et al. [2]	1986	f		Rectal cancer
Erikoglu et al. [3]	2004	f	63	Rectal cancer, 7 cm above the anocutaneous line
Bounovas et al. [4]	2007	f	85	Cancer of the sigmoid colon
McNicol et al. [5]	2008	f	72	Rectal cancer & synchronous cancer of the sigmoid colon
Chen et al. [6]	2008	f	75	Cancer of the sigmoid colon
Cetinkaya et al. [7]	2016	m	68	Rectal cancer

f, female; m, male.

In further single cases, benign lesions combined with rectal prolapse have been reported [6, 8, 9]. In particular, lipomas ranging from 3 to 8 cm [6, 8] in size were diagnosed, in a few cases, adenoma was found [6, 9] and in one subject, hamartoma in an infant was described [6].

Other authors published the coincidence of a rectal prolapse with synchronous colorectal cancer at a more oral segment of the colon, i.e., not associated directly with tumor site at the mucosa of the externalized segment of the colon [5, 10]. This clinical finding has been also described by McNicol et al. in a patient in whom two colorectal cancer lesions occurred simultaneously, namely, at the mucosa of the externalized rectum and at the not-externalized segment of the sigmoid colon [5]. Three further case reports presented rectal cancer combined with prolapse of anal mucosa, i.e., no real rectal prolapse [11], [12], [13].

For the occurrence of rectal prolapse, chronic obstipation, colonic elongation and, possibly, colon cancer are considered predisposing factors; all of them can lead to inspissation of stool by prolongation of passage time through the colon and, finally, to an insufficiency of rectal suspension.

Initially, invagination of the sigmoideorectal junction into rectal ampulla called intussusception or inner prolapse develops prior to manifestation of a prolapse of the whole colorectal wall. Subsequently, rectal wall turns to the outside leading to an external prolapse. How far there is an impact of colon cancer onto the generation of rectal prolapse is still unclear. Rashid et al. calculated in a retrospective study that in 70 patients with prolapse, colon cancer prevalence was 5.7 % whereas with no prolapse, there was only a prevalence of 1.4 % resulting in a 4.2-fold elevated cancer risk in prolapse patients [14].

In the presented case, chronic obstipation was denied. In addition, a previous prolapse episode was also not reported. But the patient had a distinct colonic elongation of the sigmoid colon segment. Furthermore, the colonic segment with malignant tumor lesion was identified to be sigmoid colon. In the published cases as mentioned above, it turned out that in two out of six patients, colon cancer was located at the sigmoid colon segment. Even if taken into account that colonic elongation was reported, it needs to be assumed that colonic elongation was present since otherwise cancer could not become apparent.

Combined with rectal prolapse and more than this with intussusception, the disease is called “solitary rectal ulcer syndrome”. Schwandner et al. showed in a retrospective evaluation of more than 1,000 patients, prevalence for solitary rectal ulcer amounts 0.7 %. In almost 50 % of – exclusively – female patients, an inner rectal prolapse could be detected whereas in a male individual, taking biopsies led finally to the detection of malignant tumor lesion [15]. This underlines the importance of histological investigation of ulcers; this includes also ulcers associated with rectal prolapse.

Due to the good physical condition of the patient and an acceptable risk-benefit ratio, anterior resection was favored instead of Altemeier procedure, a possible surgical alternative option.

### Corner points

– A 69-years old female patient with coincidence of cancer of the sigmoid colon at a manifest rectal prolapse was diagnosed.
– Literature search (using the data bank of “PubMed”) resulted in only six patients (the majority of them were females) with the simultaneous finding coincidence of rectal prolapse and rectal or colon cancer have been reported so far.
– In conclusion, a patient with a manifest rectal prolapse needs always to undergo colonoscopy and – in case of an ulcer – histological investigation of representative biopsies.
– Despite the extreme rareness, patients with rectal prolapse need to undergo always and soon complete colonoscopy to exclude malignant tumor lesion.
– An eventually coexisting ulcer requires to take biopsies for histological investigation.
